# The Impact of Gastric Atrophy on the Incidence of Diabetes

**DOI:** 10.1038/srep39777

**Published:** 2017-01-03

**Authors:** Tse-Ya Yu, Jung-Nan Wei, Chun-Heng Kuo, Jyh-Ming Liou, Mao-Shin Lin, Shyang-Rong Shih, Cyue-Huei Hua, Yenh-Chen Hsein, Ya-Wen Hsu, Lee-Ming Chuang, Mei-Kuei Lee, Ching-Hsiang Hsiao, Ming-Shiang Wu, Hung-Yuan Li

**Affiliations:** 1 Health Management Center, Far Eastern Memorial Hospital, New Taipei City, Taiwan; 2 Chia Nan University of Pharmacy and Science, Tainan, Taiwan; 3 Department of Internal Medicine, New Taipei City Hospital, New Taipei City, Taiwan; 4 Department of Internal Medicine, National Taiwan University Hospital, Taipei, Taiwan; 5 Division of Clinical Pathology‚ National Taiwan University Hospital Yun-Lin Branch, Yun-Lin, Taiwan

## Abstract

Gastric atrophy results in lower plasma ghrelin, higher gastrin secretion, a change in gut microbiota, and altered dietary nutrient absorption, which may be associated with the incidence of diabetes. *Helicobacter pylori* (*H. pylori)* infection is a major cause of gastric atrophy and is associated with diabetes in some reports. Since there is no study which investigates the impact of gastric atrophy on diabetes, we conduct a prospective cohort study to examine the relationship between *H. pylori* infection, gastric atrophy, and incident diabetes. In this study, subjects with gastric atrophy had a lower risk of incident diabetes, compared to those without gastric atrophy. The extent of gastric atrophy, measured by serum pepsinogen (PG) I/II ratio, was correlated with age, *H. pylori* IgG titer, HOMA2-IR, and HOMA2%B. When gastric atrophy is more extensive, presented as a lower serum PG I/II ratio, the risk of incident diabetes is lower. On the other hand, there was no significant association between *H. pylori* infection and the incidence of diabetes. In conclusion, the presence and the extent of gastric atrophy, but not *H. pylori* infection, are associated with incident diabetes. Further studies are needed to investigate the detailed mechanisms and the potential applications of the findings to guide diabetes screening and treatment strategies.

Diabetes mellitus is a global threat in public health nowadays. Since there is still much to be improved in the treatment of diabetes, to prevent or delay diabetes is an important strategy to fight against the diabetes epidemic. To achieve this, identification of treatable causes is a key and the first step for success.

Gastric atrophy is characterized by chronic inflammation of gastric mucosa with loss of gastric glandular cells. In practice, serum pepsinogen (PG) I and II can be used as a screening tool for gastric atrophy, defined by a low serum PG I (≤70 ng/mL) and a low PG I/II ratio (≤3). A decreased serum PG I level or PG I/II ratio not only reflects an increasing extent of gastric atrophy^1^,^2^, but also correlates with a decreased plasma ghrelin^3^, which is an important gut hormone involved in glucose homeostasis^4^. Besides, gastric acid secretion is decreased in subjects with gastric atrophy. Decreased gastric acid secretion can induce gastrin secretion^5^, change the compositions of gut microbiota^6^,^7^ and influence dietary nutrient absorption^8^. Taken together, these reports suggest that gastric atrophy may be associated with the incidence of diabetes.

Among various causes of gastric atrophy, *Helicobactor pylori* (*H. pylori*) infection is a major one. *H. pylori* infection results in various gastrointestinal disorders, such as chronic gastritis and peptic ulcer^9^,^10^. Besides, *H. pylori* infection correlates to metabolic syndrome, an important risk factor of diabetes^11^. Some epidemiological studies indicate a higher prevalence of *H. pylori* infection in patients with diabetes^12^,^13^,^14^. In cross-sectional studies, *H. pylori* infection has been associated with insulin secretion, Hemoglobin A1c (HbA1c), and diabetes^15^,^16^. There is only one prospective study which investigated the influence of *H. pylori* infection on the incidence of diabetes. In the report, *H. pylori* infection was associated with an increased incidence of diabetes in a Latino elderly cohort^17^. However, controlling for markers of systemic inflammation including C-reactive protein and interleukin-6 did not attenuate the effect of *H. pylori* infection, suggesting that factors other than systemic inflammation, such as above-mentioned changes in gastric atrophy, may contribute to the link between *H. pylori* infection and the incidence of diabetes.

To the best of our knowledge, there is no study which investigates the relationship of gastric atrophy to the incidence of diabetes. Besides, previous studies examining the association between *H. pylori* infection and diabetes did not take gastric atrophy into account, and most available data come from cross-sectional studies. Therefore, we conducted a community-based, prospective cohort study to investigate the relationship between *H. pylori* infection, gastric atrophy, and the incidence of diabetes.

## Results

At baseline, there were 1379 individuals who were not diagnosed as diabetes. Among them, 855 subjects (62%) were successfully followed. There was no significant difference between participants who were successfully followed and who were lost to follow-up (Supplementary Table 1). Among the 855 subjects who were successfully followed, 306 (36%) subjects were positive for *H. pylori* infection and 78 (9%) subjects were diagnosed as gastric atrophy at baseline. During a 3.4 ± 1.6 years of follow-up, 73 subjects developed type 2 diabetes. Table 1 presents the baseline characteristics of these subjects according to diabetes status at the end of follow-up. Compared with subjects who did not develop diabetes, those with incident diabetes were older and had higher levels of body mass index (BMI), systolic blood pressure, fasting plasma glucose, oral 75-g glucose tolerance test (OGTT) 2-h plasma glucose, HbA1c, HOMA2-IR, total cholesterol, triglyceride, low-density lipoprotein (LDL) cholesterol, and high-sensitive C-reactive protein (hsCRP).

Table 2 shows the correlations between serum PG I/II ratio with *H. pylori*-specific immunoglobulin G (IgG) antibody titers and other important risk factors of diabetes. In Pearson’s correlation tests, serum PG I/II ratio was inversely correlated with age, BMI, HOMA2-IR, and *H. pylori* IgG titer. The negative relationship of serum PG I/II ratio to HOMA2-IR and *H. pylori* IgG titer remained significant after adjusting for age and gender (p < 0.05). Neither male sex, fasting glucose, OGTT 2-h glucose, HbA1c, nor hsCRP showed any significant association with serum PG I/II ratio (all p > 0.05). Although the correlation coefficient between serum PG I/II ratio and HOMA2%B did not reach statistical significance, the partial correlation test confirmed this negative relationship after adjusting for age and gender (partial correlation coefficient *r* = −0.0778, p = 0.0233). Supplementary Table 2 shows the relationship between gastric atrophy, *H. pylori*-specific IgG antibody titers and other important risk factors of diabetes. Gastric atrophy was significantly associated with age and *H. pylori*-specific IgG antibody titers. When adjusted for age, male sex was borderline significantly associated with gastric atrophy (adjusted odds ratio 0.59 [95% CI 0.35–1.00], p = 0.050). Neither BMI, fasting glucose, OGTT 2-h glucose, HbA1c, HOMA2-IR, HOMA2%B, nor hsCRP showed any significant association with gastric atrophy (all p > 0.05).

We used Cox proportional hazards models to calculate the HRs of *H. pylori* infection, serum PG I/II ratio, and gastric atrophy to predict the incidence of diabetes (Table 3). In univariate analyses, serum PG I/II ratio was associated with the incidence of diabetes with borderline significance (HR 1.84 [95% CI 0.94–3.60], p = 0.073). Subjects with serum PG I/II ratio in the middle and the highest (borderline significance) tertile had a higher risk of incident diabetes compared with those in the lowest tertile (the middle tertile, HR 1.87 [95% CI 1.02–3.42], p < 0.05; the highest tertile, HR 1.86 [95% CI 0.99–3.47], p = 0.052). In model 1, serum PG I/II ratio was significantly associated with incident diabetes, adjusted for age, gender, and BMI (HR 2.14 [95% CI 1.12–4.11], p < 0.05). The HRs for incident diabetes in the middle and highest tertile of serum PG I/II ratio were 2.13 (95% CI 1.16–3.90, p < 0.05) and 2.15 (95% CI 1.15–4.04, p < 0.05), respectively in this model. The association between serum PG I/II ratio and incident diabetes remained significant after further adjustment for log-transformed HOMA2-IR, log-transformed HOMA2%B (model 2), family history of diabetes, HbA1c, blood pressure, and lipid profiles (model 3). For gastric atrophy, it was borderline significantly associated with incident diabetes in model 1 and was significantly associated with incident diabetes in model 2 and 3 (model 1, HR 0.35 [95% CI 0.11–1.12], p = 0.076; model 2, HR 0.28 [95% CI 0.08–0.90], p < 0.05; model 3, HR 0.28 [95% CI 0.09–0.91], p < 0.05). Serum PG I/II ratio and gastric atrophy remained significantly associated with incident diabetes when *H. pylori* infection was introduced as a covariate (serum PG I/II ratio, adjusted HR 2.21 [95% CI 1.10–4.46], p < 0.05; gastric atrophy, adjusted HR 0.29 [95% CI 0.09–0.97], p < 0.05; both adjusted for age, gender, BMI, log-transformed HOMA2-IR, log-transformed HOMA2%B, family history of diabetes, HbA1c, systolic blood pressure, diastolic blood pressure, log-transformed triglyceride, total cholesterol, and serostatus of *H. pylori* infection). In addition, inclusion of hsCRP in the model 3 did not change the effect of gastric atrophy and serum PG I/II ratio on incident diabetes (gastric atrophy, adjusted HR 0.28 [95% CI 0.09–0.92], p < 0.05; serum PG I/II ratio, adjusted HR 2.07 [95% CI 1.11–3.86], p < 0.05). On the other hand, neither *H. pylori* serostatus nor serum PG I concentrations were associated with the incidence of diabetes in univariate and multivariate models. The findings remained constant after further adjustment for gastric atrophy.

Moreover, in our study, 665 subjects had follow-up data on *H. pylori* serostatus at the last visit. Among these subjects, there were 26 subjects with *H. pylori* sero-conversion, which was defined as a change from a positive or borderline positive to a negative serologic test result. After excluding these 26 subjects with sero-conversion, there was still no significant association between *H. pylori* infection and the incidence of diabetes (HR 1.00 [95% CI 0.99–1.00], p = 0.608).

**Figure 1:**
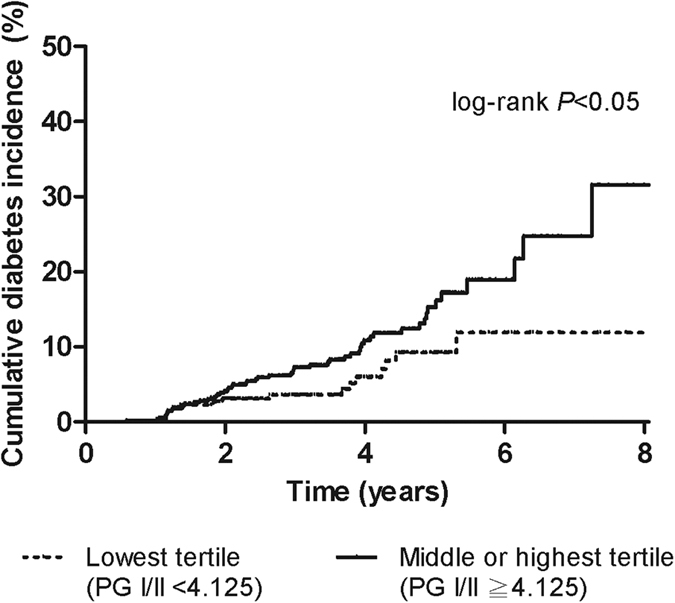
Kaplan-Meier curve of cumulative diabetes incidence by serum pepsinogen (PG) I/II ratio. Solid line, subjects in the middle or the highest tertile; dashed line, subjects in the lowest tertile.

In Fig. 1, we divided the population into two categories according to tertile of serum PG I/II ratio as follows: group 1 = the lowest tertile, serum PG I/II ratio < 4.125; group 2 = the combination of the middle and the highest tertile, serum PG I/II ratio ≥ 4.125. The Kaplan-Meier curve demonstrated that subjects with a lower serum PG I/II ratio, which means more extensive gastric atrophy, had a lower risk of incident diabetes (p < 0.05 by log-rank test).

## Discussion

In this prospective cohort study, subjects with gastric atrophy had a lower risk of incident diabetes, compared to those without gastric atrophy. The extent of gastric atrophy, measured by serum PG I/II ratio, was correlated with age, *H. pylori* IgG titer, HOMA2-IR, and HOMA2%B. When gastric atrophy was more extensive, presented as a lower serum PG I/II ratio, the risk of incident diabetes was lower. On the other hand, there was no significant association between *H. pylori* infection and the incidence of diabetes.

To our best knowledge, this is the first paper that shows gastric atrophy predicts the incidence of diabetes. In a cross-sectional study, Tanaka *et al*.^18^ reported that serum PG I/II ratio was positively related to several metabolic parameters, including plasma glucose, triglyceride, and uric acid levels. By contrast, the present study did not find a significant association between serum PG I/II ratio and plasma glucose levels at baseline. Instead, serum PG I/II ratio was negatively correlated with both HOMA2-IR and HOMA2%B. Since the improved insulin sensitivity with increasing serum PG I/II ratio may be offset by the attenuated β-cell function, the negative association of serum PG I/II ratio to HOMA2-IR and HOMA2%B can explain why serum PG I/II ratio was not associated with plasma glucose levels. In addition, we found that serum PG I/II ratio was negatively associated with age, BMI, HOMA2-IR and HOMA2%B (Table 2). In other words, the extent of gastric atrophy was positively associated with age, BMI, HOMA2-IR, and HOMA2%B, since a lower serum PG I/II ratio stands for a more extensive gastric atrophy. In Table 1, we have demonstrated that old age, high BMI and high HOMA2-IR were also risk factors for incident diabetes, which means that these factors would have confounded the relationship between serum PG I/II ratio and incident diabetes. Therefore, when these confounders were not adjusted, serum PG I/II ratio was not associated with incident diabetes (Table 1 and crude analysis in Table 3). However, when these confounders were adjusted, serum PG I/II ratio was significantly associated with incident diabetes. Similar conditions were also found for the relationship between gastric atrophy and incident diabetes.

In the present study, serum PG I/II ratio, but not serum PG I, was associated with incident diabetes. PG I is secreted by chief cells in the fundic glands^19^, whereas PG II is secreted by both fundic glands and pylogic glands^20^. In 1982, Samloff *et al*. have investigated the relationship between serum PG I, serum PG II, serum PG I/II ratio and the gastric mucosal histology^21^. In subjects with superficial gastritis (without gastric atrophy), both serum PG I and PG II were higher than normal subjects, which may result from inflammation in gastric mucosa. Since the increase in serum PG II was greater than the increase in serum PG I, these subjects had a lower serum PG I/II ratio than normal subjects. In subjects with mild-to-moderate atrophic gastritis, serum PG I was slightly decreased, reflecting the consequence of inflammation and loss of fundic glands. However, their serum PG II was still higher than that in normal subjects, which may result from pyloric metaplasia, i.e. replacement of pyloric glands for the loss of glands damaged by inflammation^22^. As a result, their serum PG I/II ratio decreased further to less than 3. In subjects with severe atrophic gastritis, serum PG I was markedly reduced, whereas serum PG II was similar to that in normal subjects. Therefore, their serum PG I/II ratio was the lowest. In Japan and other countries, serum PG I/II ratio has been used as a screening test for patients with chronic gastritis and gastric cancers^1^,^23^,^24^. Based on these reports, serum PG I/II ratio is better than serum PG I alone for defining and evaluating the extent of gastric atrophy.

The mechanism by which gastric atrophy affects the incidence of diabetes is unclear. Here, we propose three hypotheses as follows:

First, the lack of ghrelin secretion in gastric atrophy could be one possibility. Ghrelin can regulate body weight not only through its short-term effect to increase food intake^25^, but also through its long-term regulation of energy balance and increased adipogenesis^26^. In addition, ghrelin can stimulate growth hormone secretion^27^, reduce glucose-stimulated insulin secretion^28^,^29^, and induce hyperglycemia. It has been reported that plasma ghrelin levels correlated positively with both serum PG I and serum PG I/II ratio^3^. In other words, plasma ghrelin levels decreased with advancing extent of gastric atrophy. Therefore, low plasma ghrelin levels in subjects with gastric atrophy might explain why gastric atrophy predicts a lower incidence of diabetes in this study.

Second, the consequences of reduced gastric acidity may be another mechanism linking gastric atrophy to a lower risk of diabetes. Gastric acid secretion is decreased in subjects with gastric atrophy. Hypochlorhydria can induce gastrin-producing G cell to secrete more gastrin^5^. Gastrin can stimulates β-cell neogenesis and replication, and reduces β-cell apoptosis, resulting in expansion of β-cell mass and improved glucose homeostasis^30^,^31^. With the progression of the severity of gastric atrophy, there is a progressive reduction in gastric acidity and a progressive increase in luminal gastrin concentrations^32^. In this study, there was a significant inverse correlation between serum PG I/II ratio and HOMA2%B. Taken together, it is possible that gastric atrophy results in a reduced gastric acidity and a higher gastrin secretion, which expand β-cell mass and lower the risk of incident diabetes. Further studies are needed to prove this hypothesis. On the other hand, decreased gastric acid secretion can also change the compositions of gut microbiota^7^,^33^ and influence dietary nutrient absorption^8^. It is reasonable to hypothesize that gastric atrophy might decrease diabetes risk through the interaction of host and gut microbiota, which is worthwhile to be explored in future researches.

Another potential mechanism linking gastric atrophy and incident diabetes is inflammation. Gastric atrophy is the final stage of chronic gastritis, which is a multistep, progressive, and long-term inflammation^34^. Although chronic systemic inflammation can results in metabolic disturbance^35^, serum PG I/II ratio and gastric atrophy was not associated with serum hsCRP in this study. In other words, the inflammation may be confined within stomach and does not result in systemic inflammation. Indeed, inclusion of serum hsCRP, a systemic inflammatory marker, in the statistic models did not change the effect of gastric atrophy on incident diabetes. This suggests that either serum PG I/II ratio or gastric atrophy was associated with incident diabetes independent of hsCRP.

The relationship between *H. pylori* infection and diabetes remains controversial. Several studies support our findings on the lack of association between *H. pylori* infection and diabetes. It has been shown that the rate of *H. pylori* infection was similar between subjects with and without diabetes in Hong Kong Chinese population^36^. The prevalence of diabetes was not related to *H. pylori* sero-positivity after adjustment for demographic factors^37^. In contrast, Hsieh *et al*.^15^ have found that *H. pylori* infection was significantly associated with higher HbA1c and decreased insulin secretion in Taiwanese population. However, these previous reports are all cross-sectional design, the temporal relationship between *H. pylori* infection and diabetes cannot be clarified. By contrast, the present study is a prospective longitudinal cohort study. *H. pylori* infection was detected before the development of diabetes. With this design, there is no significant association between *H. pylori* serostatus and incident diabetes. Nevertheless, another longitudinal study by Jeon *et al*.^17^ has demonstrated that *H. pylori* infection leads to an increased incidence of diabetes in Latino elderly population. The discrepant findings may result from race or ethnic difference, different prevalence of *H. pylori* infection and gastric atrophy, and different age groups studied. In the Latino elderly study, 93% of the study subjects were sero-positive for *H. pylori* infection, whereas in the present study, only 36% of the study subjects were sero-positive for *H. pylori* infection. The average age of the study subjects were 68.7 years in the Latino elderly study, while the average age was 49.3 years in the present study. Besides, since there was no information on the prevalence of gastric atrophy in Latino study, we cannot compare the differences between the two studies. Taken together, further longitudinal studies from different populations are needed, in order to understand the interaction of race or ethnicity, prevalence of *H. pylori* infection and gastric atrophy, and age on the relationship between *H. pylori* infection and the incidence of diabetes.

This is the first prospective study to investigate the role of gastric atrophy on the incidence of diabetes. The longitudinal study design allows us to accurately clarify the temporal relationships between gastric atrophy, *H. pylori* infection, and the incidence of diabetes. Furthermore, *H. pylori* infection leads to chronic inflammation of stomach, and that is why *H. pylori* infection correlates with PGI/II ratio, which is a marker for gastritis. However, the extent and severity of gastritis after infection depends on interaction among *H. pylori* virulence factor, host genetic factor and environmental factor. Our study revealed that gastric atrophy, rather than *H. pylori* per se, affect the incidence of diabetes. This finding can resolve previous controversial results on the issue of diabetes and *H. pylori* infection since not every *H. pylori* infected patient will develop gastric atrophy.

By contrast, our study has some limitations. First, we do not have the information on *H. pylori* eradication, which may affect glucose metabolism and insulin sensitivity^38^,^39^. However, some patients in our study had follow-up data on *H. pylori* serostatus at the last visit. After excluding those with sero-conversion, non-significant association between *H. pylori* infection and the incidence of diabetes remained constant (see Results for detail numbers). Therefore, it is reasonable to speculate that *H. pylori* eradication has limited influence on our findings. Second, we did not measure the plasma levels of ghrelin and gastrin which have been reported to be related to both gastric atrophy and glucose homeostasis. Third, gastric atrophy may be affected by the duration of *H. pylori* infection and the severity of local inflammation. Since the duration of *H. pylori* infection is not available in this study, it remains unknown whether the relationship between gastric atrophy and the incidence of diabetes is independent to the duration of *H. pylori* infection (in order to know the independent relationship between local inflammation and the incidence of diabetes).

In conclusion, we have demonstrated that subjects with gastric atrophy are associated with a lower incidence of diabetes. When gastric atrophy is more extensive, presented as a lower serum PG I/II ratio, the risk of incident diabetes is lower. By contrast, there is no significant association between *H. pylori* infection and the incidence of diabetes. Further studies are needed to investigate the detailed mechanisms and the potential applications of the findings to guide diabetes screening and treatment strategies.

## Material and Methods

### Patients

We derived the data from the Taiwan Lifestyle Study, a large prospective cohort study^40^. From 2006 to 2012, individuals aged 18 years and above and were free of diabetes, defined as fasting plasma glucose <126 mg/dL (7.0 mmol/L) tested in the previous year at National Taiwan University Hospital Yun-Lin branch, were invited in this cohort study. All subjects were evaluated by questionnaires, undergoing physical examinations and blood tests including oral glucose tolerance tests, with the aid of trained nurses. All study subjects were contacted by telephone, e-mail, or postal mail 1–3 years after the initial visit and every 2 years thereafter. Follow-up visits were scheduled according to the respondent’s availability.

All subjects diagnosed with diabetes during follow-up were classified as type 2 diabetes by endocrine specialists based on their BMI, family history of diabetes, age, and the fact that no one had diabetic ketoacidosis. In this study, we excluded the following patients from the analysis: (1) individuals with previously diagnosed diabetes or who met the diagnostic criteria of diabetes at enrollment, (2) individuals with missing information for serum PG levels and *H. pylori*-specific antibody titers, and (3) individuals who failed to be followed. The institutional review board of National Taiwan University Hospital approved the study protocol, and written informed consent was obtained from each participant prior to enrolment. The methods were carried out in accordance with the Declaration of Helsinki ethical principles for medical research.

### Measurements and assays

Blood samples were collected from each participant after overnight fasting and then stored at −70 °C for serological assay. A standard 75-g OGTT was performed to measure fasting and 2-hour plasma glucose. Plasma glucose, lipid profiles, uric acid, and hsCRP were measured with an automatic analyzer (Toshiba TBA 120FR, Toshiba Medical Systems Co., Ltd., Tokyo, Japan). HbA1c was measured by an automatic analyzer (HLC-723 G7 HPLC system, Tosoh Corporation, Tokyo, Japan). The HbA1c assay was certified by the National Glycohemoglobin Standardization Program (NGSP) and standardized to the Diabetes Control and Complications Trial (DCCT) reference assay. Plasma insulin was measured by an automatic analyzer using microparticle enzyme immunoassay (Abbott AxSYM system; Abbott Laboratories, Abbott Park, IL). Updated computer models for homeostasis model assessment were used to calculate the indices of insulin resistance (HOMA2-IR) and insulin secretion (HOMA2%B). *H. pylori*-specific IgG antibody titers were measured using a commercially enzyme-linked immunosorbent assay assay (R-Biopharm AG, Darmstadt, Germany). Serum PG levels were measured by chemiluminescent enzyme immunoassay (Eiken Chemical Tokyo, Japan).

### Definitions

Diabetes was diagnosed by the results of OGTT and hemoglobin A1c, and if the subject was taking medications for diabetes, according to American Diabetes Association criteria^41^. *H. pylori*-specific IgG antibody titers ≥16 U/mL, 10–16 U/mL, and <10 U/mL was defined as positive, borderline positive, and negative for *H. pylori* infection, respectively. Gastric atrophy was defined as PG I ≤ 70 ng/mL and PG I/II ratio ≤ 3. PG I/II ratio was used as a measure of the extent of gastric atrophy. According to a meta-analysis^42^, the performance of using the combination of serum PG I and serum PGI/II ratio to screen gastric atrophy is good, with a sensitivity of 79% (95% CI 72–85%), a specificity of 89% (95% CI 85–93%), and an area under the ROC curve 0.87 (95% CI 0.81–0.92).

### Statistical analyses

Sample size estimation for *H. pylori* infection to predict the incidence of diabetes was done with the following data. The prevalence of *H. pylori* infection has been reported to be 50–80%^43^. The incidence of diabetes in the present cohort has been reported to be 2.92% per year^40^. In a follow-up period of 3.4 years, there will be 9.92% of the study subjects who develop incident diabetes. The hazard ratio (HR) for incident diabetes in subjects with *H. pylori* infection has been reported to be 2.69^17^. Therefore, the sample size needed to detect a difference in the incidence of diabetes between subjects with and without *H. pylori* infection at a two-sided alpha level of 0.05 and power of 80% is 324 when the prevalence of *H. pylori* infection is 50%, or 505 when the prevalence of *H. pylori* infection is 80%. Continuous variables with normal distribution were presented as means ± standard deviation (SD). Variables with skewed distribution were presented as medians (interquartile ranges) and were analyzed after logarithmic transformation. The statistical significance of the differences in different subgroups was tested with Student’s *t* test, Fisher’s exact test or *χ*^*2*^ test. Unadjusted Pearson’s correlation coefficients and partial correlation coefficients, adjusting for age and gender, were used to test the relationship between serum PG I/II ratio and other variables. Logistic regression analyses were used for the relationship between gastric atrophy and other clinical characteristics. We performed Cox proportional hazards models to estimate the hazard ratios of determinants for the incidence of diabetes. Variables significantly associated with the incidence of diabetes in univariate Cox proportional hazards models and clinically important variables were included in multivariate analyses. The incidence of diabetes in subgroups was estimated by the Kaplan-Meier method and was tested by log-rank tests. A two-tailed *p*-value below 0.05 was considered significant. Stata/SE 14.0 for Windows (StataCorp LP, College Station, TX) was used for statistical analyses.

## Additional Information

**How to cite this article**: Yu, T.-Y. *et al*. The Impact of Gastric Atrophy on the Incidence of Diabetes. *Sci. Rep.* **7**, 39777; doi: 10.1038/srep39777 (2017).

**Publisher's note:** Springer Nature remains neutral with regard to jurisdictional claims in published maps and institutional affiliations.

## Supplementary Material

Supplementary Table 1 and 2

## Figures and Tables

**Table 1:** Clinical characteristics of participants at baseline, stratified by diabetes status during follow-up.

	Participants who did not develop diabetes	Participants who developed diabetes	*P*
N (%)	782 (91)	73 (9)	
Age (years)	48.7 ± 11.9	54.9 ± 10.2	**<0.0001**
Male (N, %)	275 (35)	31 (42)	0.213
Body mass index (kg/m^2^)	23.8 ± 3.4	25.1 ± 2.7	**0.0015**
Family history of diabetes (N, %)	317 (41)	32 (44)	0.577
Systolic blood pressure (mmHg)	122 ± 16	127 ± 17	**0.003**
Diastolic blood pressure (mmHg)	78 ± 10	80 ± 10	0.1588
Fasting plasma glucose (mmol/L)	4.94 ± 0.44	5.33 ± 0.56	**<0.0001**
OGTT 2-h plasma glucose (mmol/L)	6.11 ± 0.11	7.55 ± 1.83	**<0.0001**
HbA1c (mmol/mol)	38 ± 4.4	41 ± 4.4	**<0.0001**
HbA1c (%)	5.6 ± 0.4	5.9 ± 0.4	**<0.0001**
HOMA2-IR	0.75 (0.5–1.1)	1.1 (0.84–1.43)	**<0.0001**
HOMA2%B	80.65 (62.6–104.8)	90.9 (68.6–109.5)	0.2377
Total cholesterol (mmol/L)	5.00 ± 0.91	5.34 ± 0.96	**0.0017**
Triglyceride (mmol/L)	0.99 (0.71–1.44)	1.36 (0.88–1.80)	**0.0002**
HDL cholesterol (mmol/L)	1.35 ± 0.34	1.29 ± 0.31	0.2271
LDL cholesterol (mmol/L)	3.00 ± 0.83	3.34 ± 0.96	**0.0011**
Uric acid (μmol/L)	321 ± 89	345 ± 77	0.0651
hsCRP (nmol/L)	0.76 (0.38–1.43)	1.24 (0.67–2.76)	**0.0005**
*H. pylori* IgG titer (U/mL)	5 (1.8–38.9)	4.1 (1.6–33.3)	0.5778
*H. pylori* serostatus			0.699
Negative (N, %)	462 (59)	47 (64)	
Borderline positive (N, %)	37 (5)	3 (4)	
Positive (N, %)	283 (36)	23 (32)	
PG I (ng/mL)	49.4 (39.6–65.7)	52.6 (40.6–65.4)	0.1553
PG II (ng/mL)	50.6 (38–66.4)	56.2 (43.6–71.1)	0.8807
PG I/II ratio	4.59 (3.67–5.47)	4.75 (4.21–5.57)	0.1714
Gastric atrophy (N, %)	75 (9)	3 (4)	0.139

Means ± SDs or medians (interquartile ranges) are shown.

Abbreviations: OGTT, oral glucose tolerance test; HbA1c, hemoglobin A1c; HDL, high-density lipoprotein; LDL, low-density lipoprotein; hsCRP, high-sensitive C-reactive protein; PG, pepsinogen.

**Table 2:** The relationship between serum pepsinogen I/II ratio and other clinical characteristics.

	*r*	p	partial r*	p*
Age (years)	−**0.1444**	**<0.0001**		
Male sex	0.0391	0.2530	0.0597‡	0.0810‡
Body mass index (kg/m^2^)	**−0.0691**	**0.0443**	−0.0663	0.0539
Fasting plasma glucose (mmol/L)	−0.0608	0.0756	−0.0296	0.3877
OGTT 2-h glucose (mmol/L)	−0.0418	0.2220	0.0064	0.8531
HbA1c (%)	−0.0266	0.4380	0.0242	0.4801
HOMA2-IR†	−**0.0873**	**0.0108**	−**0.0905**	**0.0083**
HOMA2%B†	−0.0560	0.1020	−**0.0778**	**0.0233**
hsCRP (nmol/L)†	−0.0269	0.4323	−0.0165	0.6303
*H. pylori* IgG titer (U/mL)†	−**0.4000**	**<0.0001**	−**0.3858**	**<0.0001**

Serum PG I/II ratio was a measure of the extent of gastric atrophy. A lower serum PG I/II ratio stands for a more extensive condition of gastric atrophy. Serum PG I/II ratio was log transformed for analysis.

Abbreviations: OGTT, oral glucose tolerance test; HbA1c, hemoglobin A1c; hsCRP, high-sensitive C-reactive protein.

^*^Adjusted for age and gender except for gender.

^†^Log transformed for analysis.

^‡^Adjusted for age.

**Table 3:** Hazard ratios (95%CIs) of *H. pylori* infection or gastric atrophy to predict incident diabetes.

	Crude	Model 1	Model 2	Model 3
**H. pylori infection**				
IgG titer§	0.98 (0.86–1.11)	0.93 (0.82–1.06)	0.90 (0.79–1.02)	0.90 (0.79–1.03)
Serostatus				
Negative	1	1	1	1
Borderline	0.89 (0.28–2.86)	0.79 (0.24–2.55)	0.93 (0.29–3.01)	0.61 (0.18–2.02)
Positive	0.82 (0.50–1.36)	0.70 (0.43–1.17)	0.66 (0.40–1.09)	0.62 (0.37–1.04)
**Gastric atrophy**				
No	1	1	1	1
Yes	0.40 (0.13–1.27)	0.35 (0.11–1.12)‡	**0.28** (**0.08**–**0.90**)†	**0.28** (**0.09**–**0.91**)†
**Serum PG I/II ratio**				
Continuous variable§	1.84 (0.94–3.60)‡	**2.14** (**1.12**–**4.11**)†	**2.35** (**1.27**–**4.35**)*	**2.06** (**1.11**–**3.84**)†
Tertile category				
Lowest	1	1	1	1
Middle	**1.87** (**1.02**–**3.42**)†	**2.13** (**1.16**–**3.90**)†	**2.34** (**1.27**–**4.32**)*	**2.36** (**1.26**–**4.44**)*
Highest	1.86 (0.99–3.47)‡	**2.15** (**1.15**–**4.04**)†	**2.32** (**1.23**–**4.38**)*	**2.36** (**1.24**–**4.49**)*

^*^p < 0.01, ^†^p < 0.05, ^‡^0.05 < p < 0.10.

^§^Log-transformed.

Serum PG I/II ratio was used as a measure of the extent of gastric atrophy. A lower serum PG I/II ratio stands for a more extensive condition of gastric atrophy. Abbreviation: PG, pepsinogen.

Model 1: adjusted for age, gender, body mass index.

Model 2: adjusted for age, gender, body mass index, log-transformed HOMA2-IR, log-transformed HOMA2%B.

Model 3: adjusted for age, gender, body mass index, log-transformed HOMA2-IR, log-transformed HOMA2%B, family history of diabetes, HbA1c, systolic blood pressure, diastolic blood pressure, log-transformed triglyceride, and total cholesterol.
