# Supplementary material for: The Impact of Gastric Atrophy on the Incidence of Diabetes
Source: Sci Rep. 2017 Jan 3;7:39777. doi: 10.1038/srep39777 (PMC5206635; doi:10.1038/srep39777)
Supplement: Supplementary Table 1 and 2 [file srep39777-s1.doc]

**The Impact of Gastric Atrophy on the Incidence of Diabetes**

Tse-Ya Yu; Jung-Nan Wei; Chun-Heng Kuo; Jyh-Ming Liou; Mao-Shin Lin; Shyang-Rong Shih; Cyue-Huei Hua; Yenh-Chen Hsein; Ya-Wen Hsu; Lee-Ming Chuang; Mei-Kuei Lee; Ching-Hsiang Hsiao; Ming-Shiang Wu; Hung-Yuan Li

Supplementary Table 1. Baseline characteristics of participants who were successfully followed and who were lost to follow-up.

|  | Participants lost to follow-up | Participants who were followed | *P* |
| --- | --- | --- | --- |
| N (%) | 524 (38%) | 855 (62%) |  |
| Age (years) | 48.2 ± 13.8 | 49.3 ± 11.9 | 0.1469 |
| Male (N, %) | 213 (41) | 306 (36) | 0.071 |
| Body mass index (kg/m^2^) | 24.1 ± 3.8 | 23.9 ± 3.3 | 0.3135 |
| Family history of diabetes (N, %) | 186 (35) | 349 (40) | 0.419 |
| Systolic blood pressure (mmHg) | 123 ± 18 | 122 ± 16 | 0.3999 |
| Diastolic blood pressure (mmHg) | 78 ± 11 | 79 ± 10 | 0.2895 |
| Fasting plasma glucose (mmol/L) | 4.97 ± 0.44 | 4.97 ± 0.44 | 0.8317 |
| OGTT 2-h plasma glucose (mmol/L) | 6.13 ± 1.66 | 6.21 ± 1.59 | 0.3941 |
| HbA1c (mmol/mol) | 38 ± 4.4 | 38 ± 4.4 | 0.5894 |
| HbA1c (%) | 5.6 ± 0.4 | 5.6 ± 0.4 | 0.5894 |
| HOMA2-IR | 0.77 (0.48-1.13) | 0.78 (0.51-1.14) | 0.4342 |
| HOMA2%B | 81.15 (61.1-103.7) | 82 (63.1-105) | 0.5205 |
| Total cholesterol (mmol/L) | 5.02 ± 0.91 | 5.01 ± 0.93 | 0.9075 |
| Triglyceride (mmol/L) | 1.02 (0.72-1.46) | 1.00 (0.72-1.50) | 0.9330 |
| HDL cholesterol (mmol/L) | 1.32 ± 0.33 | 1.35 ± 0.33 | 0.1135 |
| LDL cholesterol (mmol/L) | 3.05 ± 0.82 | 3.02 ± 0.83 | 0.5919 |
| Uric acid (μmol/L) | 330 ± 89 | 325 ± 87 | 0.4162 |
| hsCRP (nmol/L) | 0.86 (0.38-1.71) | 0.76 (0.38-1.52) | 0.7063 |

Means ± SDs or medians (interquartile ranges) are shown.

Abbreviations: OGTT, oral glucose tolerance test; HbA1c, hemoglobin A1c; HDL, high-density lipoprotein; LDL, low-density lipoprotein; hsCRP, high-sensitive C-reactive protein

Supplementary Table 2. The relationship between gastric atrophy and other clinical characteristics. Odds ratio (OR), 95% CI and p values by logistic regression analysis were shown.

|  | OR | p | Adjusted OR* | p* |
| --- | --- | --- | --- | --- |
| Age (years) | **1.03 (1.01-1.05)** | **0.002** |  |  |
| Male sex | 0.68 (0.41-1.14) | 0.145 | **0.59 (0.35-1.00)‡** | **0.050‡** |
| Body mass index (kg/m^2^) | 1.00 (0.94-1.08) | 0.905 | 1.00 (0.93-1.08) | 0.942 |
| Fasting plasma glucose (mmol/L) | 1.01 (0.98-1.04) | 0.637 | 1.00 (0.97-1.03) | 0.947 |
| OGTT 2-h glucose (mmol/L) | 1.00 (0.99-1.01) | 0.819 | 0.99 (0.99-1.00) | 0.185 |
| HbA1c (%) | 1.39 (0.74-2.61) | 0.303 | 0.97 (0.50-1.89) | 0.935 |
| HOMA2-IR† | 0.95 (0.61-1.47) | 0.805 | 0.96 (0.61-1.51) | 0.862 |
| HOMA2%B† | 0.86 (0.45-1.65) | 0.646 | 0.97 (0.50-1.90) | 0.929 |
| hsCRP (nmol/L)† | 0.95 (0.75-1.19) | 0.638 | 0.92 (0.73-1.17) | 0.505 |
| *H. pylori* IgG titer (U/mL)† | **1.55 (1.37-1.77)** | **<0.001** | **1.56 (1.36-1.78)** | **<0.001** |

* Adjusted for age and gender except for gender.

† Log transformed for analysis.

‡ Adjusted for age

Abbreviations: OGTT, oral glucose tolerance test; HbA1c, hemoglobin A1c; hsCRP, high-sensitive C-reactive protein
